# Patient, parent and professional expert perspectives on personalized regenerative implants: a qualitative focus group study

**DOI:** 10.1080/17460751.2024.2386214

**Published:** 2024-09-02

**Authors:** Manon van Daal, Anne-Floor J de Kanter, Roel JH Custers, Elena Martínez-Sanz, Annelien L Bredenoord, Nienke de Graeff

**Affiliations:** a Department of Bioethics & Health Humanities, Julius Center for Health Sciences & Primary Care, University Medical Center Utrecht, Utrecht University, Utrecht, The Netherlands; b Department of Orthopedic Surgery, University Medical Center Utrecht, Utrecht University, Utrecht, The Netherlands; c Department of Anatomy & Embryology, Faculty of Medicine, Complutense University of Madrid, Madrid, Spain; d Erasmus School of Philosophy, Erasmus University Rotterdam, Rotterdam, The Netherlands; e Department of Medical Ethics & Health Law, Leiden University Medical Center, Leiden University, Leiden, The Netherlands

**Keywords:** ethics, implant, perspectives, qualitative research, regenerative medicine, stakeholder participation

## Abstract

**Background:** Perspectives of patients, parents and professional experts on personalized regenerative implants for regenerative medicine purposes are largely unknown.

**Method:** To better understand these perspectives, we conducted four focus groups with professional experts of mixed European nationality (n = 8), Dutch patients with regular implants (n = 8), Dutch and Belgian (n = 5) and Spanish (n = 8) parents of children with cleft palate.

**Results:** Two overarching themes were identified: ‘patient-centered research and care’ and ‘ambivalent attitudes toward personalized regenerative implants’.

**Discussion:** The results reveal that stakeholders should adopt a participatory rather than an impairment discourse and address the ambivalence among professional experts, patients and parents.

**Conclusion:** Considering stakeholder perspectives facilitates ethical and responsible development and use of personalized regenerative implants.

## Abstract (plain language summary)

It is largely unknown how patients, parents and professional experts think about personalized regenerative implants.

Learning more about their perspectives is relevant because it can assist in aligning the design and implementation of these implants with the needs and desires of stakeholders such as patients, engineers and clinicians.

The first overarching theme that emerged from the focus groups was the importance of patient-centered research and care. Three subthemes were identified: (a) support and expectation management, (b) institutional and interdisciplinary collaboration, (c) the implant’s contribution to living one’s life.

A second theme explored in the focus groups concerned participants expressing ambivalent attitudes toward the characteristics of personalized regenerative implants, such as their regenerative capacity, the novelty of this technology and the possibility for personalization.

The first overarching theme patient-centered research and care underscores the necessity for healthcare professionals to look beyond the mere technical and clinical aspects of personalized regenerative implant treatment.

In medicine in general and regenerative medicine in particular, we should facilitate a shift toward participatory discourse and change the way we talk and approach the individual, their body and illness.

Some of the ambivalent attitudes identified in this study were linked to characteristics of the implant itself, particularly personalization. Personalization was viewed as a potential contributor to improved care tailored to individual needs, alongside concerns about associated cost increases.

During the development and use of personalized regenerative implants, researchers should address ambivalence among professional experts, patients and parents, keeping in mind that ambivalence is not inherently negative.

The obtained results provide a valuable first step for emphasizing the need for patient-centered care and research within regenerative medicine and exploring ambivalent attitudes toward personalized regenerative implants.

Further evaluation of these insights and other ethically relevant aspects of personalized regenerative implants should take place in co-creation with diverse stakeholders in parallel to the technological development of these implants.

## 1. Introduction

A shift is taking place in the field of medical implants. For decades, off-the-shelf and inert implants, like metal and plastic implants for knee and hip replacements (hereafter referred to as regular implants) were primarily used. Recently, a new generation of implants has emerged for tissue engineering and regenerative medicine [1–3]. These implants are regenerative, because they facilitate cell growth by serving as a scaffold (artificial extracellular matrix) for tissue repair and regeneration. Once a physiologically and structurally functional tissue has formed, these implants typically biodegrade, meaning that they break down into nontoxic by-products and leave the body after a certain period. These implants can also be personalized to fit the patients’ bodies and are mostly created using additive manufacturing. Personalized regenerative implants are currently being developed for healthcare applications including the treatment of musculoskeletal disease. The focus of this paper will be on cleft palate and joint defects.

Currently, cleft palate is treated through a series of surgeries performed between birth and adulthood (around 18 years old), involving palatoplasty and recurrent insertion of ventilation tubes in the ear, followed by subsequent surgeries addressing issues like maxillary closure, speech problems or aesthetic concerns [4]. During these surgeries, autogenous bone grafting is commonly used for bone regeneration. Personalized regenerative implants offer a promising alternative, growing with the child’s development and potentially streamlining the process to a single surgery. Also, they hold the potential to achieve closure of both hard and soft palates using a combination of materials.

Current treatments for joint defects involve nonregenerative implants or autologous, allogenic and xenogenic bone grafting often lacking patient-specific dimensions, regenerative properties, stable materials, regular surfaces (compared with native tissue) and mechanical stability [5]. Personalized regenerative implants, like an osteochondral or meniscus implant for the knee joint, present a potential solution, tailoring the implant to the individual, reducing surgery duration, ensuring mechanical stability, fostering tissue regeneration, prevent shrinkage and allowing the use of multiple materials simultaneously.

To use personalized regenerative implants responsibly in healthcare, the ethical issues that play a role in implant development and use need to be understood. Existing literature has identified various ethical issues related to inert regular implants and regenerative medicine applications, including tissue engineering and biofabrication [6–10]. Recent reviews raised awareness of underexplored ethical issues of tissue engineering for regenerative purposes, including irreversibility, the inclusion of gender differences and embodiment, with only one of these reviews focusing on personalized regenerative implants [11,12]. Moreover, qualitative studies have been published in which professional experts share their insights on the ethical aspects of regenerative medicine applications in orthopedics and congenital birth defects in children, all involving the use of stem cells [13–15]. Important ethical themes in these studies related to potential serious adverse effects, like the increased risk of cancer, the source of cells and the cell donation process [14,15]. However, issues related to the use of stem cells such as source of cells and donation process are not relevant for cell-free implants, which are the central focus of this paper. Additionally, multiple qualitative studies have explored patients’ experiences with regular implants, including total knee replacements and parents' perspectives on cleft palate services [16–19]. The themes that are discussed within these studies are related to the lived experiences of implant recipients, such as embodiment, bodily sensations, psychological factors and interpersonal skills of healthcare professionals [16–19].

While there is qualitative literature on how professional experts think about ethical aspects of regenerative medicine applications (including the use of stem cells) in different fields and how patients and parents of children think about regular treatments for joint defects and cleft palate, it is largely unknown how these patients, parents and professional experts think about personalized regenerative implants. Learning more about their perspectives is relevant because it can assist in aligning the design and implementation of these implants with the needs and desires of stakeholders such as patients, engineers and clinicians.

This paper presents the results of a focus group study into the perspectives of patients, parents and professional experts on personalized regenerative implants. These insights are linked to the results of previously published literature. By doing so, this analysis contributes to the responsible development and implementation of newly emerging technologies in regenerative medicine, while also providing practical guidance for the implementation of these technologies in real-world scenarios.

## 2. Methods

In this study, we investigated professional experts’, patients’ and parents’ perspectives on personalized regenerative implants through a qualitative focus group study. Focus groups are a qualitative research method to gather a comprehensive understanding of attitudes, opinions and experiences within a specific context [20]. The interaction among participants is used to obtain broader and deeper information than is possible in an individual interview. This focus group study is part of the INKplant consortium, which seeks to develop personalized regenerative implants for tissue engineering and regenerative medicine using different biomaterials, high-resolution additive manufacturing and advanced simulation and biological evaluation. The study is reported following the consolidated criteria for reporting qualitative studies guidelines [21].

### 2.1. Participant selection & recruitment

The participants of this study were divided into three different groups of stakeholders (Table 1). The first group [n = 8] were patients in the Netherlands who will get or have already gotten regular inert synthetic shoulder, knee or hip implants. Among this group, two patients underwent experimental treatments involving the use of biological materials or stem cell therapy, while the remaining six participants received state-of-the-art implants. The second group (n = 13) were parents whose children had undergone current state-of-the-art treatment options, including various surgeries performed from birth to adulthood (around 18 years old) to treat cleft palates and maxillary openings. This group was divided into two focus groups: one with parents from the Netherlands and Belgium (n = 5) and the other with Spanish parents (n = 8). The last group (n = 8) were orthopedic surgeons, biomedical engineers and researchers from different European countries. These professional experts all worked in the field of regenerative medicine and tissue engineering and had extensive knowledge of personalized regenerative implants.

**Table 1.** Respondent groups.

	Patients (the Netherlands) (n = 8)	Parents (Spain) (n = 8)	Parents (the Netherlands and Belgium) (n = 5)	Professional experts (Europe) (n = 8)
**Type of implant** Regular Semiregular	6 2	n/a n/a	n/a n/a	n/a n/a
**Type of cleft palate** Cleft lip and cleft palate Cleft palate	n/a n/a	5 1	5	n/a n/a
**Gender (participants)** Man Woman	5 3	3 5	5	5 3
**Birth sex (child)** Man Woman	n/a n/a	6 2	4 1	n/a n/a
Age participant Age child	33–77 years n/a	n/a 2–15 years	n/a 2–11 years	n/a n/a
**Profession** Biomedical engineer Surgeon/clinician	n/a n/a	n/a n/a	n/a n/a	5 3

Dutch patients were recruited via R. Custers’s network of clinicians in the University Medical Center Utrecht and via Dutch patient organizations ‘Reuma Nederland’ and ‘Osteoporose Vereniging’ and their social media channels. Dutch parents of children with cleft palate were recruited via Schisis Nederland and their social media channels. Spanish parents of children with cleft palate were recruited via E. Martínez-Sanz together with clinicians from two hospitals in Madrid (Gregorio Marañón University Hospital and Hospital Infantil Universitario Niño Jesús). Professional experts were recruited via the European INKplant consortium and the Dutch Materials Driven Regeneration consortium. Both consortiums work on regenerative implants for tissue engineering.

The research protocol was submitted to the research ethics committee of the University Medical Center Utrecht for review before starting the research. The committee determined that this study was exempt from the Medical Research Involving Humans Act (research proposal no. 21/828). In line with the research protocol, we obtained written informed consent from all participants.

### 2.2. Data collection

Focus groups were conducted between March 2022 and March 2023. The focus groups lasted between 90 and 120 min. All focus groups took place online via MS Teams due to covid-19 restrictions, climate change considerations and accessibility reasons. The focus groups were conducted in Dutch, English or Spanish. The Spanish focus group was moderated by E. Martínez-Sanz and observed by N. de Graeff. The other focus groups were moderated by M. van Daal and observed by A. de Kanter or N. de Graeff. The topic list of the focus groups was based on a previous review of the ethical aspects of personalized regenerative implants [11,12] and discussions among the research team and the INKplant consortium. The interviews consisted of open-ended questions related to hopes, expectations, potential benefits and risks and potential ethical implications of the personalized regenerative implants. These questions allowed participants to bring up or emphasize specific issues they considered relevant, while also ensuring some consistency in the topics that were discussed to explore how different participants viewed these topics. As is typical of qualitative research, the topics evolved during the focus groups. The participants were in the lead of the conversation. The focus groups were recorded, transcribed verbatim, translated into English (when necessary) and pseudonymized.

### 2.3. Data analysis

Data from this study was analyzed thematically. In general, data analysis was based on the constant comparative method, which involves reviewing the data to develop to develop codes, concepts and themes by continually revisiting the data for both supportive and conflicting evidence [22]. M. van Daal and A. de Kanter coded the full transcripts by labeling units of texts that referred to one or more topics relevant to the study. Nvivo 12 software was used for coding. Afterward, M. van Daal and A. de Kanter compared and checked the codes for consistency and critically discussed the interpretations and suitability of the codes. The codes were adjusted across transcripts. After consensus on coding was reached, the codes were developed into higher-order concepts and themes and discussed among all authors. This process was iterative by repeatedly revisiting the data. Representative quotes were chosen to illustrate the patients’, parents' and professional experts' experiences and translated into English by M. van Daal.

## 3. Results

The analysis of the results of the focus groups have led us to identify two overarching themes: (1) patient-centered research and care and (2) ambivalent attitudes toward personalized regenerative implants. These overarching themes and related subthemes were discussed by participants in different ways, as we will discuss below. Not all subthemes were discussed in all focus groups.

### 3.1. Theme 1: Patient-centered research & care

The first overarching theme that emerged from the focus groups was the importance of patient-centered research and care. Patients and parents of children with cleft palate underlined points of improvement regarding the care they had received thus far on the one hand and wishes they had with regard to the care surrounding future regenerative implants, on the other hand. Professional experts also identified points for improvement for patient-centered research. Three subthemes were identified: (a) support and expectation management, (b) institutional and interdisciplinary collaboration, (c) the implant’s contribution to living one’s life.

#### 3.1.1. Support & expectation management

Patients with regular implants expressed a need for a better support system during their treatment and recovery journey. Patients emphasized effective communication and the importance of recognizing the myriad challenges stemming from their comorbidities, as important elements of their support. Some patients indicated that they did not always experience the acknowledgement of these challenges and that better communication would have significantly helped them throughout their treatment journey. However, parents of children with cleft palate praised the extraordinary support they received from their healthcare professionals. Parents even referred to them as their ‘cleft palate family’ (Table 2, quote 1).

**Table 2.** Illustrative quotes about patient-centered research and care.

Support	
1	DP3: Yes, from, or after the palate closure I believe, I don't remember exactly, but since about six years we meet annually or biennially with those involved in the cleft palate team to sit at the table with us as parents and the child. So the next steps are indeed taken in a multidisciplinary manner and are also discussed with you present. So yes, it does kind of feel like your “cleft palate family” taking care of your child. I also have a lot of confidence in it and the communication is good. – *Dutch parent*
2	SP7: No, P5 raises a very interesting subject because in the end we all know it well, cleft palate is not the same, there are as many cases as there are children, right? So in the end, regarding the success rate it would be important to consider it because, we do not know, right? No, it is not the same unilateral, bilateral and there are a thousand, a billion cases, so (…) if the professional could guide us on the success rate of doing it in one way or another, I think it would be very important, because from there, as P5 says, you can decide, right? So look, in the traditional method you have ninety percent and they never tell you because they can't tell you, right? But a very high percentage or ‘look it is the best option you have because the case is very complicated and and we think this is the best option for your son’, well, then you can evaluate it. – *Spanish parent*
3	P2: And yes, when looking at myself, my knee has been open four-times and yes, like [P5] says: at some point you make a decision, like when you can no longer walk or there’s pain every day, then it is worth opening it up again. However, it doesn't get any better and you know that as well. After the first time, it wasn't too bad, but then every next time new issues arise, such as scar tissue and irritation. So the threshold is getting higher and higher. And then I think; maybe if I had been guided much better in the preliminary phase by speaking to this group of people, or by being informed much better on the consequences, then that might have saved me one or two operations. Because then you will really start to think more seriously and perhaps make the better choice at the start of things, or not make it and postpone it for later, in order to make a better choice 3 years later for instance. So I think there is a lot to be gained, especially in the preliminary stages. – *Patient*
4	P2: We've all been through one or more surgeries and after, you find out that things aren't as promising as you thought they would be, or as it was presented to you. And in my experience, with regard to the preparation, in the preliminary phase, I think there should be much more attention on what awaits you and a more realistic picture should be drawn concerning all the side effects and additional ailments, that entire rehabilitation process actually. This way you are able to consider it properly. (…) Even providing some mental support could be helpful if necessary, according to most people (from what I hear), it is in fact disappointing. It’s just much more complex and you encounter a lot more things than you expected, which also affects you mentally. – *Patient*

Institutional and interdisciplinary collaboration	
5	P6: Well, there needs to be more of a collaboration with and between the specialists about what the patient’s issues are. Different professionals have to respond to this and together they have to make a plan for you, or for us. And that’s the point, none of that is carried out, because they are all rulers in their own field. And “Ms this or Ms that”, but they don't truly feel you. – *Patient*
6	P7: Well, what I find unfortunate is that I'm being treated at [hospital 1] for one thing, at [hospital 2] for another and undergoing surgery again on my back in [hospital 3]. And they can't receive or request each other’s data. Because they work with different systems. So, I have to give permission again to one, because otherwise, they are not allowed to forward it to the other. And then they say: just take it on a disk, because that works even better. I think that’s so completely old-fashioned. – *Patient*
7	DP2: Yeah, gosh, all I can say is that I think it’s fantastic, really. Depending on what stage we were at, we saw only certain doctors, but once a year we also had something called a cleft palate consultation. And then the entire team is present, comprised of an orthodontist, a dentist, the head of the team, the surgeon who will perform the next operation, a psychologist, a speech therapist, a nutritionist. (…) So yes, that was very nice and convenient as well, because as a parent you don't always… sometimes so much is said, you don't remember everything or you don't fully understand it all. The fact that they communicated a lot among themselves and that they were often all present during a consultation, communicating with each other, while you, as a parent, were present, I found that very pleasant. Because you feel like you're always informed about what’s being discussed. – *Belgian parent*
8	DP6: But what I still find very particular is that, regarding cleft palates, there are still so many hospitals that all work with different approaches. (…) And I wonder whether [a regenerative] implant can soon be implemented in all hospitals. And the same goes for the age limit of the operation. In our [name hospital] we simply went 100 percent for closing the soft and hard palate at the same time to avoid further surgery. (…) for me the question arises (…) do hospitals even agree with each other on how they're going to perform cleft palate surgeries, before we start using a [regenerative] implant? – *Dutch parent*
9	EP3: I think, we, as scientists, we tend to stay too close to our, to what we know. – *Professional expert*
10	EP7: It’s good to (…) have the other stakeholders have a look and give their estimation on the value, as well. Because everyone looks at it from their own perspective, they see their value. But it’s, yeah, I, I see that for myself. I try to think from the patient and maybe from the orthopedic surgeons' point of view. But I'm not a patient and I'm not an orthopedic surgeon. So, I just try to think myself into that position. That one should really collaborate on a deep level with the people that actually have this expertise. Whereas we still often do our own work from our perspective and publish it this way. – *Professional expert*

Living one’s life	
11	P7: (…) after many internal issues in the hospital, they decided to give me a new knee implant 8 years ago (…). It went well. And I must say that I didn't experience any problems afterward. I can walk again, however, I can't sit on my knee, but the doctor says: why do you need to be able to sit on one knee? I say, ‘Well not to pray, doctor, you can do that in all kinds of positions, but just to grab something for instance’. But, like I said, that doesn't work. And I've been alone for a very long time, but you find solutions for everything. – *Patient*
12	P7: And now a year ago, I received a reverse shoulder because I had two torn tendons. (…) And I have to say, if I had known beforehand, I wouldn't have done it. It limits me a lot, I can't move my arm any further than this. I had to remodel my entire house to be able to reach everything (…) and different clothing and so on. Because you can no longer do things yourself, like putting on something with a zipper. Anyway, the doctor says: then you would have kept the pain. But I say: now I have a different kind of pain, if I lift it higher than I should, it hurts. Anyway, I try to adapt and live with it, but this is not, this doesn't make it easy, the reverse shoulder. – *Patient*
13	DP4: Yes, I can imagine that it can be an improvement on a psychological level if it can be limited to one or two surgeries or at least as few as possible. Because it can be traumatic, the fact that it all happens in this area. It concerns eating and well how [SP2] just described it, how challenging and intense that can be. I also experienced that when she was very little, unfortunately. Refusing to eat. Eating, is of course, a form of control that you can have when you have no control because surgeries have to take place. So, fewer traumas, fewer psychological concerns, could be an advantage and a consequence. – *Dutch parent*
14	DP2: For me, driving back and forth every time to [city] is an issue. There have been periods, especially leading up to the last surgery, when we had to go to [city] weekly or bi-weekly. Each time he had to miss a day of school. That is, well, he is now behind in reading and writing, for example. He is catching up, but those things could be avoided if the last surgery is dismissed. So I think that’s really important to take into account. – *Belgian parent*

Parents and patients emphasized the significance of expectation management as a crucial element of presurgical support. Various parents explained that they received valuable tips and insights from clinicians and other healthcare professionals, enabling them to be better informed about the upcoming surgery and its challenges. They also highlighted that providing this comprehensive information about what to expect from surgery and the need for clear guidance was important for future treatments with personalized regenerative implants (Table 2, quote 2). Some patients who had already received regular implants expressed that the medical team had fallen short in terms of expectation management in the care they had received. Several patients had been given certain expectations that led to disappointments during their treatment journey. For instance, one patient highlighted that if they had been better informed and guided in the beginning, they possibly could have avoided some of the surgeries and could have made a more informed choice about whether to undergo another surgery or not (Table 2, quote 3). For many patients, the treatments turned out to be more complex than anticipated and the outcomes of these treatments were disappointing, resulting in mental and emotional impacts. Patients highlighted that it was important to them that the medical team draws a realistic picture (regarding all possible side effects, complications and the rehabilitation process) and offers them emotional guidance to navigate the emotional challenges of the treatment process. This would enable the patients to make informed decisions about future treatments with personalized regenerative implants (Table 2, quote 4).

During the professional expert focus group, the involvement of patients and the public in research was mentioned as a way to manage the expectations of these groups. For instance, professional experts highlighted that engaging with patients and the public through interactive workshops to explain research goals, methodologies and setting realistic timeframes helps to bridge the gap between scientific research and the patients and the public and ensures they are well-informed throughout the process.

#### 3.1.2. Institutional & interdisciplinary collaboration

Patients and parents emphasized that institutional collaboration sometimes lags behind (Table 2, quote 5). For instance, one patient shared a personal experience about their treatment with regular implants which had failed due to the transfer to another hospital, resulting in missed injuries or lesions and consequently, incorrect treatment. Some patients received treatment from different hospitals for various issues and did not have access to all their medical information, leading to challenges in receiving optimal care (Table 2, quote 6). Therefore, patients emphasized the significance of increased collaboration among healthcare professionals across different disciplines within hospitals and beyond to improve the quality of (new) treatments such as regenerative implants. The professional experts also acknowledged that institutional collaboration should be improved.

Parents of children with cleft palate also reported a lack of such interdisciplinary collaboration between institutes, but generally expressed positive experiences with collaboration among various healthcare professionals in different fields within the same hospital. They valued the consultation meetings held once or twice a year, involving all stakeholders, including the orthodontist, dentist, psychologist, speech therapist, nutritionist and surgeon. The fact that the healthcare professionals communicated extensively with each other in front of the parents was helpful (Table 2, quote 7). However, parents questioned the variety of approaches taken by hospitals, including variations in the age at which the first cleft palate surgery is performed and differences in methods for closing hard and soft palates (Table 2, quote 8). They encouraged hospitals to align approaches and work together both nationally and internationally to improve the implementation of new treatments such as regenerative implants.

In addition, professional experts acknowledged that they are inclined to solely operate within their specific research domains (Table 2, quote 9) and view the research process from their own perspectives by focusing on finding the best measurement for their research objectives and gather extensive data on their research topic. Professional experts mentioned three elements that could help mitigate the tendency to only operate within specific research domains and thereby contribute to interdisciplinary collaboration and an integrated research approach in the field of regenerative medicine. First, practicing empathy and gaining a deeper understanding of the experiences and needs of these stakeholders can significantly contribute to the research outcomes. For instance, one professional expert emphasized the importance of stepping into the shoes of the patients and orthopedic surgeons (Table 2, quote 10). Second, they emphasized the importance of adopting a forward-thinking approach right from the start of their research projects. For instance, one professional expert mentioned that when developing a new material for 3D printing, it is crucial to also consider the clinical aspects to ensure its applicability in real-world settings. The professional experts proposed a shift toward a more entrepreneurial mindset, where researchers have a well-defined product in mind early on and work toward that goal. Third, professional experts suggested investing in collaborative approaches, such as multidisciplinary consortia, conducting focus groups together and facilitating interdisciplinary meetings. By gathering input from different professional experts, researchers could gain valuable insights, especially those who are not part of a multidisciplinary consortium and/or working on more fundamental projects.

#### 3.1.3. Living one’s life

Certain patients expressed that healthcare professionals focused too much on rectifying a defect (e.g. a damaged shoulder) and sometimes overlooked their broader wishes and needs. Patients shared experiences of interacting with their clinicians in which the clinician focused primarily on the (physical and technical) functioning of their implant. For example, when a patient expressed the desire and perceived need of being able to sit on her (implanted) knee, her physician questioned the necessity of being able to do so (Table 2, quote 11). She explained her independent living situation, emphasizing her wish to move freely. This example sheds light on how clinicians may sometimes overlook or fail to fully comprehend the desires and needs expressed by their patients. To prevent this, patients emphasize the importance of healthcare professionals listening attentively and delving deeper into the personal experience of living with a specific disease or condition.

Furthermore, patients expressed that it was important to them that the implant would enable them to live their lives to the fullest. For instance, patients raised important questions regarding their post-treatment abilities, expressing concerns regarding to what extent they would regain the ability to walk their dog, run or swim after undergoing regenerative implant treatment. The discussions around regenerative implants prominently revolved around the concepts of independence and mobility, highlighting the need for freedom of movement and concerns about the potential limitations thereof. The ability to move independently was viewed as beneficial in supporting both physical well-being and personal fulfillment. A patient expressed that had they known beforehand about the extent to which their movements would be restricted by the (regular) implant, they might have chosen not to undergo the treatment (Table 2, quote 12).

In line with this, parents expressed a desire for their children to experience a more uninterrupted childhood, with decreased emotional and physical suffering experienced by their children as a result of the surgeries and rehabilitation. Correspondingly, they considered the potential that regenerative implants might offer for fewer surgeries as beneficial for their children as well as themselves (Table 2, quotes 13 and 14).

### 3.2. Theme 2: Ambivalent attitudes toward personalized regenerative implants

A second theme explored in the focus groups concerned participants expressing ambivalent attitudes toward the characteristics of personalized regenerative implants, such as their regenerative capacity, the novelty of this technology and the possibility for personalization. Interestingly, the ambivalent relationship with regenerative implants was also reflected in the metaphors and language patients used to talk about them. Finally, the significance of age as an important selection criterion for regenerative implants was a matter of contrasting perspectives and this divergence in views was particularly pronounced between patients and professional experts.

#### 3.2.1. Regeneration

The regenerative capacity emerged as a topic of interest in all focus groups, with participants expressing both the advantages and disadvantages of regenerative implants. This ambivalence is reflected in two ways, namely in how they talked about tissue growth and about the fixation of the regenerative implant in the body.

Regarding growth, one important benefit, as highlighted by several participants, is the possibility of the implant leading to the tissue growth and replacement of the implant by the body’s own tissue. This was perceived as advantageous since the body takes over, which one parent described as a ‘natural’ good solution for their child (Table 3, quote 1). Additionally, some participants expressed confidence that this regenerative aspect would benefit the recovery and healing process. In contrast to these benefits, parents also expressed concerns regarding the process of tissue growth and its potential implications. They wondered whether the implant and tissue would grow adequately with the child’s body as he or she ages, ensuring a functional palate in the long run. Furthermore, there were apprehensions about the possibility of uncontrolled tissue growth, such as tumor formation, as a potential risk associated with the regenerative aspect of the implant. Participants sought clarification on whether the tissue growth would stop at a certain point and how it would be controlled (Table 3, quote 2). One parent mentioned feeling uneasy about the idea of something growing inside their child’s body but also mentioned that they could get used to this idea over time (Table 3, quote 3).

**Table 3.** Illustrative quotes about ambivalent relationship with regenerative implants.

Regeneration	
1	SP10: (…) She had to go through two operations because [during] the first one (…) there was really nothing to take hold of, right? Because it was such a huge fissure that maybe one of these solutions that goes along with the growth, it closes the fissure and makes everything more natural, in a more natural way, it could have been, it would have been a super good solution for [name child]. – *Spanish parent*
2	DP4: And someone also said it and I thought about it too, how long, how far does it continue to grow? Won't it suddenly at some point, well I don't have any knowledge of it, but where does it somehow stop growing, or something? – *Dutch parent*
3	DP5: And yes, the fact that that tissue becomes one, so to speak, it is broken down and it [re]generates, I find that myself also a bit of a strange idea or something. It’s something that you really have to get used to, that it is, in a way, actually doing something in the child’s body, so to speak. – *Dutch parent*
4	EP5: (…) Second, as you all know and pointed out, it’s not the same cartilage. So, [you] might as well transplant something else. Which is you know, synthetic. There are metal options. So, you can actually implant a little button in the hole. But that has not worked well. Because the fixation is really bad. So, the bone behind it dissolves. And so, the metal then, sort of, gets loose in the joint. Which doesn't, doesn't work very well. And then, you have applications in multiple joints. Equally, if you can recreate a tendon, we have a massive problem in the rotator cuff in the shoulder. So, I think the potential [of personalized regenerative implants] is, is definitely there. (…) – *Professional expert*
5	P3: Well, I think the regenerative aspect is perhaps more of a disadvantage, that it gets absorbed by your body. You see, if you just have an implant that’s fixed, it won't wander through your body. If it gets absorbed, those cells have to go somewhere. It is something that is not in your body. Are there any studies on what will happen with this? Could it, for example, clump together in, I don't know, your lymph nodes, for example? That’s a possibility. Could it trigger a reaction? Could it encapsulate? These are all questions that I think are very important to have thoroughly investigated. – *Patient*

Novelty	
6	DP6: I think it looks really awesome and I am, well, of course I find already that there, I think that new techniques are cool anyway, just like putting a pig’s heart into humans, so I'm already enthusiastic about it. – *Dutch parent*
7	P5: That to me, sounds like music to my ears, because 3D and custom made in a mold, whether or not with an MRI, I don't know exactly how it all comes together, but that sounds fantastic. – *Patient*
8	DP5: Personally I think, you do insert something, which I personally would find a bit scary, regarding the experimental nature of it. So something that, well, that is not yet, with which there is no experience yet, the fact that you do that with your child, well, to put it strangely, your child becomes a bit of a guinea pig, so to speak. – *Dutch parent*
9	SP5: For, for him, so, well, no, we would not mind because we trust [name doctor] blindly, for us she is, she is a magician, she has directly performed magic with, with our son, eh and and if she tells us look, these are the first ones but we are sure that, that it will go well, it has good results that have already been shown, we would do it of course. – *Spanish parent*

Personalization	
10	EP4: Since all kind of palatal defects are completely different in shape. So, when we talk about personalized, I think, it’s really helpful. So that the implant fits perfectly into the defect size. It can be bilateral or unilateral. Or complete defect or partial defect. Yeah. So, this would be really helpful when it completely fits. – *Professional expert*
11	SP7: I think it is important and then the issue that I pointed out and here is the issue of equity, that is, this is something that, it will be funded or not funded, right? Because ethically it is also somewhat interesting and, well, if only those who can pay for it can access it or not, well, it should be taken into account and that’s all. – *Spanish parent*
12	EP5: The Dutch are very keen cyclists. So are Belgians. There is a saying in road cycling: “Cheap, light and strong. Pick two”. So, you need to, you can only have two out of three. So, we can't be cheap, complex and perfect. So, if it can't be cheap. At least, it has to be better than what’s biologically available. And I think, this applies to this perfectly. – *Professional expert*

Metaphors and language	
13	EP6: Yeah. I think that, of course, some of this reminds me of the repair of a car. If you're going to be of this and it has to come out working. And I think, maybe we would have to sort of change the way we treat these things. – *Professional expert*
14	P6: Take an old car, you can install a new part and the others start wearing out faster too. And that’s how I see our bodies too. – *Patient*
15	P2: My knee has been opened up four-times in 2.5 years. (…) At first, you think, well, just cut it open and fix it. – *Patient*
16	P5: I can imagine that with a 3D-printed, well yes, you want new cartilage to grow, but the big problem is that you have to insert it somewhere. And for that, you often have to open up the knee or the shoulder or the hip. And if there were something that could put it in the right place, say, by means of encapsulation, then you wouldn't need the recovery from opening that knee, shoulder or hip anymore. That would be really great for me because, well, the recovery takes so much time: the skin is cut open, but there are also several layers underneath, you have to get to the right place, it is exposed and therefore susceptible to bacteria and such. – *Patient*

Age and ageing	
17	P5: But everything is subject to age. And I don't want people to discriminate against me because I am almost 55 years old: which means it is no longer possible. Anyway, I will also have to deal with the fact that as I get older, my body may no longer recover as quickly and as good/effectively. And it is about finding balance and one body is not the same as the other, one is stronger than the other and some still recover at an old age. – *Patient*
18	P6: It has to do with wear and tear and with that other lady too, we have been living a bit longer. So yes, it is only logical that there will be defects. – *Patient*

Regarding fixation, professional experts noted that regenerative implants are appealing because of their integration into the body’s ‘natural’ structure and therefore are more securely anchored in the body than regular implants, such as metal implants (Table 3, quote 4). In contrast, one patient argued in favor of an implant that is fixed and not regenerative because it feels stable within their body and less likely to move around (Table 3, quote 5). The patient cited examples of breast prostheses and meshes that migrated through the body and caused health problems to emphasize their preference for fixation. In connection to this, another patient expressed concerns about the perceived fragility of regenerative implants and feared that it might be susceptible to breaking.

#### 3.2.2. Novelty

The novelty of the regenerative implant technology evokes both excitement and uncertainty among participants. Patients and parents expressed enthusiasm for the advanced 3D-printed implant, seeing it as incredible advancement (Table 3, quotes 6 and 7).

However, alongside this excitement, participants expressed concerns about the experimental nature of regenerative implants, particularly when it involves implanting them in a child. Patients and parents worry about the potential unknown side effects and approach it with a certain level of caution, as they would not want themselves or their children to be treated as guinea pigs (Table 3, quote 8). Three factors were mentioned that could reduce their concerns. First, the need for testing was discussed as an important element by patients and parents of children with cleft palate. Participants express their desire to know about previous testing, the outcomes and potential consequences. Older patients, in particular, do not want to take unnecessary risks and emphasize the importance of gathering more experience with regenerative implants. Second, trust in the medical professionals plays a significant role in the willingness to embrace this novel technology. This was mainly discussed by the parents in the Spanish focus group, who appeared very trusting of their clinician’s opinion on regenerative implants and showed less hesitancy toward these implants (Table 3, quote 9). They argued that if the clinician said it was trustworthy and that this treatment added value, they would let their children undergo this (experimental) treatment. Third, given the novelty of regenerative implants, both professional experts and patients emphasized the need for long-term evaluation of implant performance and outcomes, both positive and negative. Therefore, proper evaluation mechanisms are essential to assess the long-term effectiveness of these treatments accurately. Another reason for proper evaluation mechanisms pointed out by professional experts was that patients often do not communicate their concerns or dissatisfaction with their treatment to their surgeon directly but on patient forums only.

#### 3.2.3. Personalization

The idea of personalizing regenerative implants led to discussion in all focus groups. Especially in the professional expert focus group there was discussion about what personalization *is* and *how* implants should be personalized. A professional expert raised the question of how we should define personalization and which parameter should be personalized. Numerous parameters were explored during these discussions, including the external and internal structure of the implant, pore size, surface characteristics, mechanical properties, biological factors and even the patient’s lifestyle. Furthermore, professional experts underscored gender as a crucial factor in personalizing regenerative implants.

Personalization was recognized as an advantage and disadvantage. On the one hand, personalization was mentioned as a benefit because it can be tailored to a particular individual with specific needs (Table 3, quote 10). This was also endorsed by patients and parents. A professional expert suggested that personalized implants could influence patients’ attitudes. For example, upon recognizing the treatments' personalization, they may be more inclined to cooperate with the treatment and understand that part of the success of the treatment is in their hands (and could for instance be affected by quitting smoking).

On the other hand, personalization was mentioned as a disadvantage because it makes implants more complex and therefore drives up the costs. This also reinforced the concerns that were expressed regarding the accessibility of regenerative implants. Both professional experts and patients questioned whether the implants would be accessible to everyone or only to those who could afford them (Table 3, quote 11). Two factors were mentioned that could mitigate this contradiction. First, it was mentioned that a balance should be achieved between the wishes of all stakeholders, because not all needs can be met within one implant design (Table 3, quote 12). Second, a professional expert suggested that there is a need to look at the long-term cost-effectiveness of regenerative implants. This professional expert explained that the implants will be expensive in the beginning but when it reduces morbidities and surgeries in the long-term, it will be more cost-effective.

#### 3.2.4. Metaphors & language

Participants in all focus groups used reductive language, metaphors or terminology when discussing personalized regenerative implants. On multiple occasions, respondents made comparisons between the body and a car, highlighting similarities in terms of maintenance and repair (Table 3, quote 13). This metaphorical language served to conceptualize and discuss medical interventions within a familiar framework. For example, an older patient used a body-as-car metaphor to explain the aging process of her body (Table 3, quote 14). Similarly, patients used phrases such as ‘cutting open’ or ‘opening up’ when referring to surgeries and fixing bodily issues (Table 3, quotes 15 and 16).

In these instances, participants seemed to reduce their bodies to entities requiring repair. This way of speaking about their bodies was remarkable given that both patients and parents also stressed that effective treatment is about much more than merely fixing one’s body – it should lead to a fulfilling life aligned with their personal goals. The data thus seem to reflect an ambivalence: on the one hand, they discuss their bodies and the treatments in reductionist terms, viewing them as entities requiring repair, while on the other hand, they emphasize the need for treatments that respect their broader wishes and needs and align with their individual life goals.

#### 3.2.5. Age & ageing

Age was frequently mentioned during the focus groups with professional experts and patients, with these different stakeholders bringing up contrasting views. On the one hand, age was mentioned as a necessary selection criterion for regenerative implants. In the expert focus group, there was discussion about age-related regenerative capacity and who would (not) be eligible for such implants. Children were identified as an eligible user group for regenerative implants due to their need for tailored and individualized solutions. Moreover, it was noted that elderly patients with osteoarthritis, who predominantly suffer from joint degeneration, do not experience significant growth or regeneration, suggesting that these patients would benefit less from personalized regenerative implants. In contrast, another expert suggested that older individuals receiving personalized implants may experience improved healing and fewer complications compared with traditional metal implants. Within the expert focus group, children were identified as an eligible user group for regenerative implants due to their need for tailored and individualized solutions.

On the other hand, patients had different opinions about using age as a selection criterion for regenerative implant treatment. Some participants struggled with fears of age discrimination because of their older age (Table 3, quote 17). At the same time, they also stressed the importance of accepting their natural aging process (Table 3, quotes 17 and 18).

## 4. Discussion

As far as we know, this is the first study exploring the perspectives of patients, parents and professional experts on regenerative personalized implants. Although these stakeholders generally welcomed the introduction of such implants into regenerative medicine interventions for multiple several musculoskeletal applications, they addressed the importance of patient-centered research and care (theme 1) and also expressed an ambivalent attitude toward these implants (theme 2).

This first exploratory study gives an impression of the relevant themes to set the further research agenda for the regenerative medicine and tissue engineering field. In what follows, the implications and relevance of this qualitative study are considered, the findings are linked to the broader literature and underexplored topics are highlighted.

### 4.1. Insights for research & care for the broader healthcare domain

Our study results reveal insights that are relevant for the broader healthcare domain. This is mainly reflected in the first theme on patient-centered care and research and related subthemes of support, institutional and interdisciplinary collaboration, living one’s life and metaphors and language.

### 4.2. Beyond technical & clinical aspects of personalized regenerative implants

The first overarching theme patient-centered research and care underscores the necessity for healthcare professionals to look beyond the mere technical and clinical aspects of personalized regenerative implant treatment. It is crucial for them to recognize the significance of addressing the broader impact of the medical intervention on patients’ lives. In the wider literature on patient-centered research and care, this is often referred to as a holistic approach – a tendency to acknowledge the person’s entire life [23]. Coordinated care is also cited as a crucial element in this approach, involving coordination across the health system and interprofessional collaboration [23], which aligns with elements discussed under the subtheme of institutional and interdisciplinary collaboration. In the literature on implants, particularly on knee/joint implant treatments, the importance of a holistic approach is also emphasized [17]. Moore et al. [17] further suggest that if a patient encounters challenges with incorporating an implant, clinicians should engage in sensitive communication and actively listen to those experiencing such difficulties.

Through support, active listening, recognition and validation of patient concerns, as well as the integration of their experiences into the decision-making process, healthcare providers can deliver more patient-centered and effective care. This will help to bridge the gap between the clinical perspective and the patient’s lived experience and might lead to better outcomes and increased patient satisfaction with treatments with personalized regenerative implants.

The key to a patient-centered approach is to address the purpose of the treatment. The data of this focus group underline that when healthcare providers and their patients are not aligned in their treatment goals, this can result in adverse treatment outcomes, miscommunication and dissatisfaction with the treatment. Therefore, establishing a shared understanding of treatment objectives is essential for ensuring a successful and patient-centered treatment.

However, such shared understanding of the main treatment objectives, whether involving regular implants or personalized regenerative implants, is not yet well-established. In this study, patients and parents emphasized the significance of both regular and personalized regenerative implants in enabling recipients to lead fulfilling lives, indicating this as the main treatment objective. At the same time, patients perceived that their clinicians were primarily focused on fixing the defect. Additionally, explicit discussions about the treatment objective were not observed among the professional experts, who mainly concentrated on clinical and technical aspects of personalized regenerative implants. Other qualitative studies with experts did touch upon the primary goals of treatment with regenerative medicine applications. For example, in the study of Niemansburg et al. [14] experts observed that the field should engage in a thoughtful deliberation regarding the goal of regenerative medicine – whether it should primarily focus on tissue regeneration or prioritize the improvement of patients’ symptoms and overall well-being.

### 4.3. The impairment discourse versus the participatory discourse

These two perspectives on the goals of regenerative medicine applications such as personalized regenerative implants are intricately connected to the discourses surrounding illness and treatments. Bunzli et al. [24] reflect on two common discourses in discussions on knee osteoarthritis: the impairment discourse and the participatory discourse.

The impairment discourse centers on the limitations of individuals due to their condition, treating the body as a machine [24]. Rooted in mind-body dualism, it perceives the body as a physical object requiring specialized ‘mechanics’, like orthopedists, to fix breakdowns, with pain alleviated only through repairing damaged body parts [24]. In contrast, the participatory discourse emphasizes individuals' capabilities despite their limitations [24]. Clinicians aim to empower patients, valuing an active body and considering the social context’s role in shaping illness experiences rather than solely focusing on fixing defects (such as the knee joint) [24]. This approach acknowledges that while anatomical changes may be beyond an individual’s control, individuals can influence modifiable social factors impacting their health. It advocates for informed choices promoting an active lifestyle and healthy aging, recognizing that some may lack the ability, skills, confidence or resources for such decisions [24].

The impairment discourse is reflected in the data from this study at two levels. First, patients frequently mentioned that healthcare professionals tended to overlook their broader desires and needs, focusing primarily on rectifying physical defects. The focus of these healthcare professionals is in line with the impairment discourse. The impairment discourse is also recognizable in the fields of medicine and regenerative medicine more generally, where it is common to see the body as an object that can be repaired, restored or replaced [25,26]. However, the body is also a subject and the center of human experience, as has been explored extensively by philosophers, particularly in the phenomenological tradition [27,28]. This entails a stronger focus on the day-to-day experiences of patients and their relationship with their bodies. Correspondingly, an important next step for future research is empirically exploring what it is like to live with personalized regenerative implants. Thus far, this has been difficult due to the limited number of individuals currently living with such implants. Therefore, a theoretical exploration of available empirical phenomenological literature on experiences with existing implants might serve as a valuable first step in this research endeavor.

Second, the impairment discourse is reflected in the use of reductive language, metaphors and terminology within the focus groups. Professional experts frequently employ such language and focus on technical and clinical aspects when discussing patients’ bodies and patients and parents adopt similar language when addressing their (or their child’s) illness and treatments. These linguistic expressions reduce the body to an object and aligns with the idea that the body can be mechanically repaired or altered. While these phrases may be practical in conveying the nature of medical procedures, they may also contribute to an objectification of the body by reducing it to a mere entity in need of physical modifications and may thereby draw attention away from the broader wishes and needs of patients.

At the same time, the results of our focus groups show that there is an implicit desire among patients and parents to shift toward a participatory discourse. This shift is reflected in the data in multiple ways. For instance, parents articulated a desire for less disruption in their children’s lives. Patients often center their discussions around what they can achieve after receiving a personalized regenerative implant, like swimming or walking their dog, reflecting the participatory discourse’s focus on capabilities before and after treatment. Additionally, they express a desire for clinicians to understand what it means to live with a particular disease or defect. Within this discourse, a healthy joint is one that facilitates an active life throughout one’s lifespan and it does not necessarily imply a complete cure. Thus, it becomes important to gain deeper insight into treatment and research objectives that all stakeholders have in mind regarding personalized regenerative implants.

Facilitating this shift in discourse is important as adhering to the impairment discourse can have negative implications [24,29]. First, this discourse has the potential to discourage individuals from actively engaging in their own care, fostering a greater dependence on clinicians for body repair [24]. Second, clinicians who predominantly use an impairment approach may overlook the need to encourage active management strategies or offer psychological support for the failing body machinery of individuals [29]. Thus, in medicine in general and regenerative medicine in particular, we should facilitate such shift in the way we talk and approach the individual, their body and illness.

### 4.4. Insights for personalized regenerative implants & regenerative medicine

Our study also reveals insights that are specifically relevant for regenerative implants and regenerative medicine applications more broadly. This is particularly reflected in the second overarching theme of ambivalent attitudes toward personalized regenerative implants. This ambivalence echoes similar sentiments expressed by stakeholders in the literature concerning new (bio)technology in medical healthcare. For instance, a study exploring clinicians' perspectives on artificial intelligence as a medical decision aid revealed a clear ambivalence in the data [29]. Clinicians perceived artificial intelligence as a job threat but also acknowledged the potential benefits for patient care [30].

There is no consensus on the consequences of ambivalence in technological change processes [30,31]. Some argue that ambivalence should be either avoided or resolved and that successfully addressing ambivalence is a crucial step in adapting to technological change [30]. It is argued that it can lead to confirmation bias and various forms of resistance to change, including defensive reactions, noncooperative behavior or active resistance [30,31]. However, this perspective is evolving, with scholars now also considering ambivalence as a potential facilitator of positive outcomes [30]. For instance, ambivalence may foster openness to change, adaptability and positive attitudes toward minority groups [30,31].

Some of the ambivalent attitudes identified in this study were linked to characteristics of the implant itself, particularly personalization. Personalization was viewed as a potential contributor to improved care tailored to individual needs, alongside concerns about associated cost increases. A similar ambivalence was found in the literature between the perceived benefits of personalized medicine (improved healthcare and health benefits) and its risks (increased health insurance costs and uncertainty about coverage of personalized medicine) [32]. One potential explanation for this ambivalence may stem from the lack of clarity in the meaning of personalization. In this study, as well as in the literature [32], there appeared to be varying interpretations of what personalization entails. Conducting follow-up research to explore whether a clearer understanding of personalization could mitigate ambivalent attitudes toward aspects of personalized regenerative implants would be a valuable next step for investigation.

To date, we have not encountered similar ambivalence within the field of regenerative medicine. The exploration of how various stakeholders discuss the possibilities and challenges of personalized regenerative implants in our study provides valuable insights for the ongoing development and use of these implants. These insights are crucial for enhancing communication with patients and parents, guiding decisions in consent procedures and determining whether to opt for a personalized regenerative implant. This encompasses for example aspects such as the growth and fixation of the implant and the diverse experiences that different stakeholders have with this characteristic.

While our study has identified ambivalence in discussions surrounding personalized regenerative implants, the reasons for this phenomenon and its specific effects in this context remain unclear. Understanding why and when ambivalence arises are critical research endeavors to fully develop the potential of personalized regenerative implants. During the development and use of personalized regenerative implants, researchers should address ambivalence among professional experts, patients and parents, keeping in mind that ambivalence is not inherently negative.

### 4.5. Limitations

The results of this study should be interpreted within the context of several limitations. First, the scope of the study was relatively broad. Given that this was the first qualitative study on the perspectives of patients, parents and professional experts regarding personalized regenerative implants, it allowed respondents to raise issues they deemed relevant. Further research is needed to delve more deeply into these topics such as the embodied experience of recipients of personalized regenerative implants. Second, any qualitative study is influenced by the positionality of the moderators and researchers; a different moderator and researcher might have focused on different aspects of the respondents’ answers and categorized the codes and themes differently. Third, the focus groups were conducted in various languages, including Dutch, Spanish and English, with transcripts subsequently translated into English when necessary. During the translation process, there is a potential for information loss or misinterpretation of words. Fourth, the focus groups included participants predominantly born, raised and living in Europe, reflecting a mainly European perspective. Conducting similar studies in different global regions may yield different views and opinions on personalized regenerative implants. Finally, not all input provided by patients and parents of children with cleft palate is directly related to personalized regenerative implants, as none of them or their children had such implants themselves. Nevertheless, the themes they addressed hold relevance for the current field of regenerative medicine, offering valuable best practices and highlighting critical elements requiring attention or improvement in the development and use of personalized regenerative implants.

## 5. Conclusion

It is expected that the use of personalized regenerative implants in healthcare will increase in the near future. While these implants have a range of potential benefits, they also raise important challenges. This study helps to understand the perspectives of professional experts, patients and parents of patients on the ethical aspects of personalized regenerative implants. The obtained results provide a valuable first step for emphasizing the need for patient-centered care and research within regenerative medicine and exploring ambivalent attitudes toward personalized regenerative implants. Further evaluation of these insights and other ethically relevant aspects of personalized regenerative implants should take place in co-creation with diverse stakeholders in parallel to the technological development of these implants. In this way, these considerations can inform the development and implementation of personalized regenerative implants and facilitate their ethical and responsible development.
